# Magnetic nanoparticle hyperthermia for treating locally advanced unresectable and borderline resectable pancreatic cancers: the role of tumor size and eddy-current heating

**DOI:** 10.1080/02656736.2020.1798514

**Published:** 2020-12

**Authors:** Anilchandra Attaluri, Sri Kamal Kandala, Haoming Zhou, Michele Wabler, Theodore L. DeWeese, Robert Ivkov

**Affiliations:** a Department of Mechanical Engineering, School of Science, Engineering, and Technology, The Pennsylvania State University – Harrisburg, Middletown, PA, USA;; b Department of Radiation Oncology and Molecular Radiation Sciences, Johns Hopkins University School of Medicine, Baltimore, MD, USA;; c Department of Mechanical Engineering, Whiting School of Engineering, Johns Hopkins University, Baltimore, MD, USA;; d Department of Oncology, Johns Hopkins University School of Medicine, Baltimore, MD, USA;; e Department of Materials Science and Engineering, Whiting School of Engineering, Johns Hopkins University, Baltimore, MD, USA

**Keywords:** Hyperthermia, magnetic nanoparticles, pancreatic cancer, eddy currents, tumor size, bioheat transfer

## Abstract

**Purpose:**

Tumor volume largely determines the success of local control of borderline resectable and locally advanced pancreatic cancer with current therapy. We hypothesized that a tumor-mass normalized dose of magnetic nanoparticle hyperthermia (MNPH) with alternating magnetic fields (AMFs) reduces the effect of tumor volume for treatment.

**Methods:**

18 female athymic nude mice bearing subcutaneous MiaPaCa02 human xenograft tumors were treated with MNPH following intratumor injections of 5.5 mg Fe/g tumor of an aqueous suspension of magnetic iron-oxide nanoparticles. Mice were randomly divided into control (*n* = 5) and treated groups having small (0.15 ± 0.03 cm^3^, *n* = 4) or large (0.30 ± 0.06 cm^3^, *n* = 5) tumors. We assessed the clinical feasibility of this approach and of pulsed AMF to minimize eddy current heating using a finite-element method to solve a bioheat equation for a human-scale multilayer model.

**Results:**

Compared to the control group, both small and large MiaPaCa02 subcutaneous tumors showed statistically significant growth inhibition. Conversely, there was no significant difference in tumor growth between large and small tumors. Both computational and xenograft models demonstrated higher maximum tumor temperatures for large tumors compared to small tumors. Computational modeling demonstrates that pulsed AMF can minimize nonspecific eddy current heating.

**Conclusions:**

MNPH provides an advantage to treat large tumors because the MION dose can be adjusted to increase power. Pulsed AMF, with adjusted treatment time, can enhance MNPH in challenging cases such as low MION dose in the target tissue and/or large patients by minimizing nonspecific eddy current heating without sacrificing thermal dose to the target. Nanoparticle heterogeneity in tumors remains a challenge for continued research.

## Introduction

Pancreatic cancer is the fourth leading cause of cancer death in the USA, with a 5-year survival rate of less than 10%, among the lowest for all cancers [1]. Approximately 30% of patients present with unresectable loco-regional cancer and have a 5-year survival rate of 13% [2]. Treatment methods for localized or metastatic pancreatic cancers are well defined as surgical resection and chemotherapy (CT), respectively. Conversely, treatment recommendations for locally advanced (LAPC) and borderline resectable pancreatic cancer (BRPC) is unclear [3]. Standard-of-care for unresectable LAPC and BRPC includes chemo-radiotherapy (CRT) or CT alone. CRT is associated with significant toxicity and typically only temporarily stabilizes tumor progression, with only a small subset of patients (10–15%) exhibiting an objective response [4–10]. Desmoplasia, tumor microenvironment and low blood flow to the tumor are major contributors to chemoresistance in LAPC [11–13]. Local control, that is, primary tumor burden, is a crucial factor causing symptoms that are often difficult to manage and contribute to degraded quality of life [14,15]. Preoperative treatments such as stereotactic body radiation therapy (SBRT) and intensity-modulated radiation therapy (IMRT) have shown promise to aid local control and debulk or downstage the tumor to reduce the involvement of adjacent critical tissues and improve the likelihood of potentially curative surgical resection [14–21]. The proximity of radiation-sensitive organs such as duodenum, stomach and small bowels limit treatment options with SBRT and IMRT [22–26].

Tumor size is a key prognostic feature for LAPC and BRPC treatment affecting survival [26–32]. Large tumor volumes generally limit treatment options, define dose-limiting toxicities, and are associated with decreased survival [26,33]. Volumetric parameters such as metabolic tumor volume and total lesion glycolysis measured by fluorodeoxyglucose positron emission tomography/computed tomography (FDG-PET/CT) are associated with progression-free survival and overall survival in patients with LAPC [32,34–37]. There is an unmet need for additional therapeutic tools that can be used to enhance the effectiveness of current therapies for LAPC.

Hyperthermia (40–45 °C) given concurrently with CRT is being reexamined as a combination treatment to improve local control of many solid cancers [38,39]. Initial clinical studies demonstrated feasibility with results that showed reduced toxicity and improved efficacy, even in patients initially diagnosed with a poor prognosis [38]. Hyperthermia is recognized as a potential complementary treatment to sensitize tumors to CRT because it increases blood flow, oxygenation to the tumor, and inhibits DNA damage repair by tumor cells [40,41]. Tumors exhibiting low blood perfusion are generally good candidates for hyperthermia because heat transfer out of tumor to normal tissues due to blood perfusion is minimized, thereby concentrating energy within the tumor. Conventional hyperthermia techniques (such as radiofrequency, capacitive and high-intensity focused ultrasound face challenges for controlled heating of large tumors located deep within the body [42–44].

Magnetic nanoparticle hyperthermia (MNPH) is a treatment modality that exploits interactions of magnetic fields with magnetic materials to generate heat, predominantly *via* magnetic hysteresis loss [45]. MNPH received regulatory approval by the European Medicines Agency to treat recurrent GBM in combination with RT in 2010 [46]. MNPH received IDE approval from the U.S. FDA in 2018 to conduct prostate cancer clinical trials [47]. For MNPH, the region containing the magnetic iron-oxide nanoparticles (MIONs) is exposed to an alternating magnetic field (AMF). Low frequency (<10 MHz) AMFs are essentially not attenuated by tissue and thus penetrate deep into the body. Like other EM energy, intensity depends on the distance from the applicator surface. Advances in image-guided interventions, such as X-ray computed tomography (CT)/ultrasound-guided percutaneous transabdominal or endoscopic ultrasound-guided (EUS) transgastric/transduodenal delivery methods enable precise delivery of MIONs into LAPC and BRPC [48]. Generally, MIONs embedded within pancreatic tumors remain for at least 7 days in preclinical animal models [49,50], enabling multiple treatment sessions by remote activation with an AMF. Repeated MNPH treatment after a single MION injection has been clinically demonstrated in prostate and brain tumors [51–55]. Thus, MNPH offers the potential for well-controlled and repeated heating of deep tissues by controlling AMF power to modulate the heat sources embedded in the tumor [56,57].

Interactions of AMF with electrically conducting (diamagnetic) bodies, such as human tissues, induce eddy currents, that is, Faraday’s Law of induction, which deposits Joule heat to tissues [58–60]. The nonspecific eddy current heating depends on the AMF amplitude (*H*), frequency (*f*), and varies along with the radial distance (*r*) of the sample, *P*_*non*–*specific*_ ∝ σ_*t*_(*rfH*)^2^, where σ_*t*_ is the electrical conductivity of the idealized tissue. Clinically permissible limits for a 30 cm diameter region of tissue, that is, the torso is $H\times f<4.85\times 10^{8}\frac{A}{m\cdot s}$ [58]. For a successful MNPH treatment, magnetic hysteresis loss power deposited by the MIONs, *P*_*MION*_ > *P*_*nonspecific*_, and is controlled by AMF adjustments using temperature to ensure adequate thermal dose [56,57]. Eddy current heating can be reduced by limiting the area of exposure to high-amplitude AMF, as well as reducing amplitude or duty. AMF coils generating homogeneous AMF in the target region reduce nonspecific tissue heating compared to currently available clinical MNPH systems by reducing the requirement for increased amplitude at the surface to compensate for reduced amplitude at the target [61]. Thus, coils generating homogeneous AMF will reduce superficial heating in deep tissue applications such as treating LAPC. Eddy current heating can be further reduced by keeping the coil in constant motion relative to the tissue [59]. Pulsed AMF, that is, reduced duty cycle, dissipates heat generated by eddy currents by enabling physiological thermoregulatory processes to dissipate heat as demonstrated in a mouse model exposed to high-amplitude AMF [62].

Local control of large tumors with CRT presents challenges, whereas the physics of heat deposition and control with AMF, and heat transfer by physiological thermal regulation provide advantages for MNPH to enhance treatment of LAPC and BRPC. In this study, we sought to investigate (a) the potential for MNPH to reduce the effect of tumor volume on treatment using small animal and computational models; and, (b) assess using computational modeling how well pulsed AMF can manage eddy current heating for large tissue volumes. We demonstrated in a pilot study of mice growing large (0.3 cm^3^) and small (0.15 cm^3^) subcutaneous human pancreas tumors that MNPH generated comparable effects on tumor growth irrespective of tumor size. Our human-scale computational modeling showed that MNPH provides an advantage to treat large tumors. Pulsed AMF minimizes nonspecific eddy current heating without sacrificing thermal dose to target even in challenging clinical scenarios. To achieve equivalent thermal doses with pulsed power, treatment time was increased to compensate for reduced duty.

## Materials and methods

### Cell lines and reagents

Human pancreas cancer cell lines, MIA PaCa02, were purchased from American Type Culture Collection (ATCC^®^ CRM-CRL-1420^™^, Manassas, VA). According to directions by the vendor, cells were cultured in Dulbecco Modified Eagle’s Medium (DMEM) supplemented with 10% fetal bovine serum, 2.5% horse serum, and kept in a humidified incubator at 37 °C with 5% CO_2_.

### Magnetic iron oxide nanoparticles (MIONs)

MIONs (NanoMaterials Technology, Singapore) used in this study were citrate-stabilized dense polycrystalline core MIONs [58–64]. Briefly, MIONs were prepared by high-gravity controlled precipitation with thermal aging and stabilized with citric acid. Their physical, magnetic, and heating characteristics have been previously described in detail [63,64]. The stock aqueous suspension of MIONs with a concentration of 47.1 mg Fe/ml was used in this study.

### AMF system

**Figure 1.**
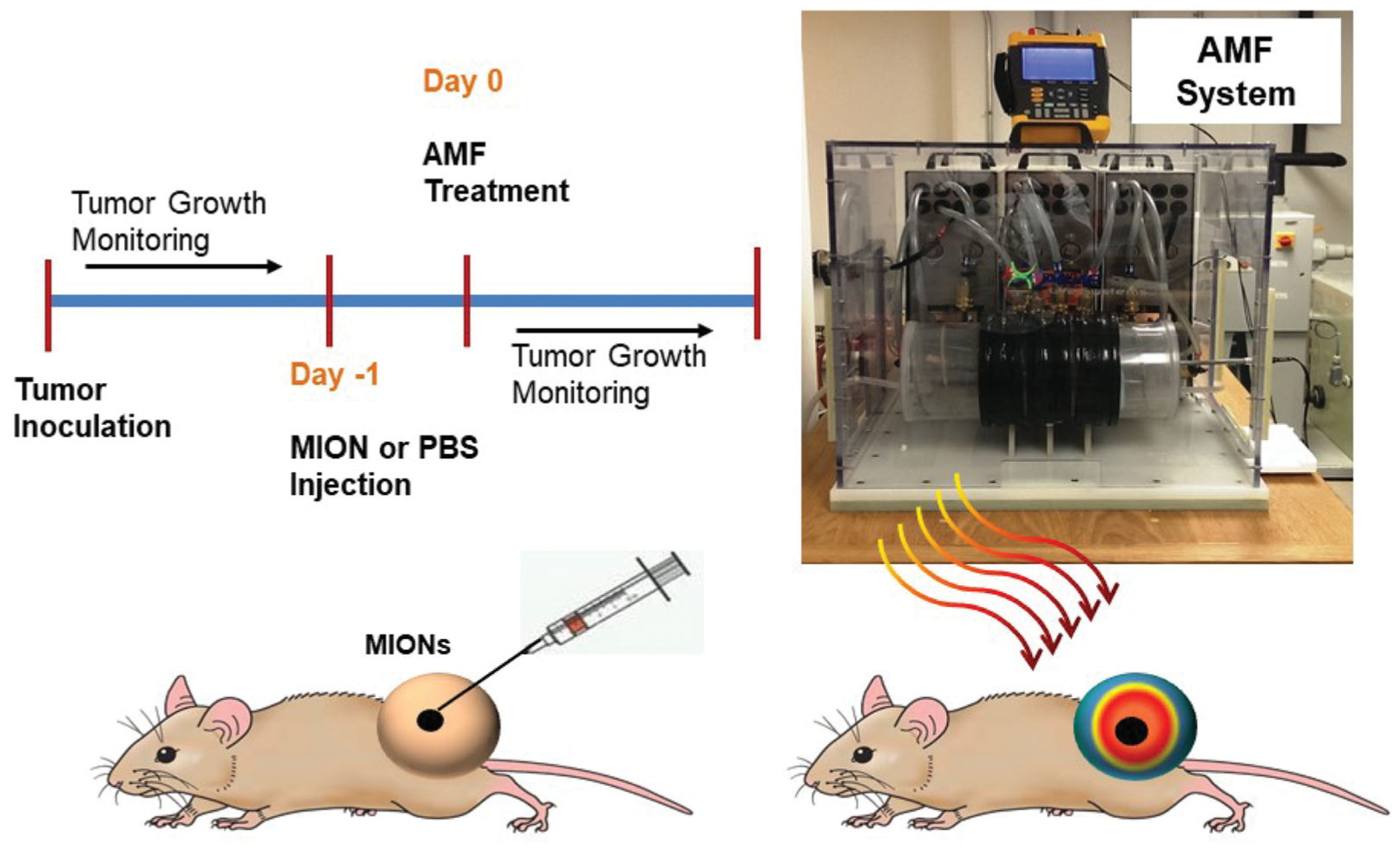
Schematic of the study design for the magnetic nanoparticle hyperthermia (MNPH) therapy with intratumor injection of magnetic iron-oxide nanoparticles (MIONs) for MiaPACa-02 tumors in mice and photograph of the alternating magnetic field (AMF) system used to perform MNPH treatments in mouse tumors.

A photograph displaying the experimental set-up (AMF system, water jacket) is shown in Figure 1. Previously described, the AMF system comprised three main components: a power supply, an external impedance matching network, and a modified Maxwell coil [61]. The AMF coil comprises three independent loops capable of generating uniform AMF amplitudes in a cylindrical volume of ≥2.7 × 10^3^ cm^3^ at 160 ± 10 kHz [61]. Prior to experiments, the magnitude of the magnetic field was measured at the multiple locations with the AMF coil using a magnetic field probe (AMF Life Systems, Inc., Auburn Hills, MI) for several power settings to provide calibration for all studies [57,61,65,66].

### In vivo xenograft experiments

The Institutional Animal Care and Use Committee (IACUC) at Johns Hopkins University approved all animal studies and were conducted using 18 female athymic nude mice (Harlan Labs, IN, USA) aged 6–7 weeks and weighing 16–22 g at beginning of study. Female nude mice were selected based on the vendor’s guidelines on the growth of MiaPaCa02 tumors. All mice were fed a normal diet and water *ad libitum*. They were maintained in the normal 12 h of light and dark. Mice were monitored closely for any distress or pain throughout the study period. A schematic of the xenograft tumor study design is provided in Figure 1.

#### Tumor inoculation and measurements

MiaPaCa-02 xenografts were obtained by injecting ~5 × 10^6^ MiaPaCa02 cells mixed 1:1 with Matrigel on the right thigh. Tumor volume was estimated from caliper measurements in three orthogonal dimensions and assuming hemi-ellipsoid geometry, *V*=0.5236 × Length × width × height, prior to injections, and mice were randomly assigned to one of treatment or control groups when tumors measured to a predetermined volume.

#### MION injection

Aliquots of the stock MION suspension diluted to ~23.6 mg Fe/ml were used as an intra-tumor injection of MIONs. Intraperitoneal injection of ketamine (0.1 mg/kg body mass)/xylazine (0.01 mg/kg body mass) preceded all animal MION injections and AMF exposures. Complete sedation of the animal was determined by a lack of response after compressing the hind paw. A three-site injection with a 25-gauge needle was performed to achieve a total delivered dose of MIONs equaling 5.5 mg Fe/cm^3^ of the tumor. For mice in the control groups, PBS or MIONs were injected using similar methods and approximately similar volumes.

#### Control and experimental groups

To study the influence of tumor size on the tumor volume-normalized MION dosed MNPH treatment, mice bearing small (0.15 ± 0.03 cm^3^) and large (0.3 ± 0.06 cm^3^) tumors were randomly assigned to control (MION-no AMF or PBS-AMF), and AMF (18 kA/m peak to peak, 160 ± 10 kHz, for 20 min) groups. Experimental cohorts each contained 4 to 5 mice. Large tumor size was decided to adhere to the IACUC tumor burden (mass of tumor (g)/Weight of the animal (g) × 100) criteria when the animal reached the study endpoint of 4 times initial tumor volume.

#### Selection of AMF settings

AMF settings were selected to achieve therapeutic hyperthermic temperatures (41–45 °C) in both small and large tumors based on pilot ex-vivo studies. AMF settings were optimized to avoid ablative temperatures (>46 °C) in large tumors. AMF settings were confirmed by measuring intra-tumor temperatures in a surrogate cohort of two mice (one small tumor and one large tumor) with MiaPaCa02 subcutaneous xenograft tumors. AMF-compatible fiber-optic temperature probes were used to measure rectal, (intra) tumor (probe tip in the approximate center of the tumor), the skin of thigh contralateral to the tumor, and water jacket temperatures.

#### AMF exposure

Twenty-four hours after MION or PBS injection, mice were anesthetized and exposed to AMF. Multiple (*n* = 2–4) mice were exposed to AMF simultaneously by placing them at the center of the water jacket inside the coil with a uniform magnetic field. Each mouse was placed in a modified 50 ml conical tube holder. Each mouse holder was placed in an acrylic tube to provide separation between the mice in a modified 50 ml conical tube holder. The water jacket temperature was set to maintain the mice’s rectum temperature in the physiological range. Styrofoam^®^ endcaps with access to temperature probes were placed at the end of the water jacket to avoid convective heat transfer with the surroundings. Only rectal temperatures were measured in a cohort of mice used for tumor growth delay studies. Tumor growth was measured by post MNPH treatment. Time to reach fourfold initial volume (*t*_0_ = time at treatment) was the chosen endpoint.

### Statistical analysis

Tumor quadrupling times were chosen as the time to endpoint (TTE). Statistical analysis and graphic presentations were conducted using GraphPad Prism 6.0 (GraphPad Software, La Jolla, CA). Non-parametric Log-rank test was used to analyze the significance of the differences between TTE of treated and control tumor groups, with differences, deemed significant at *p* < 0.05.

### Computational simulation

**Figure 2.**
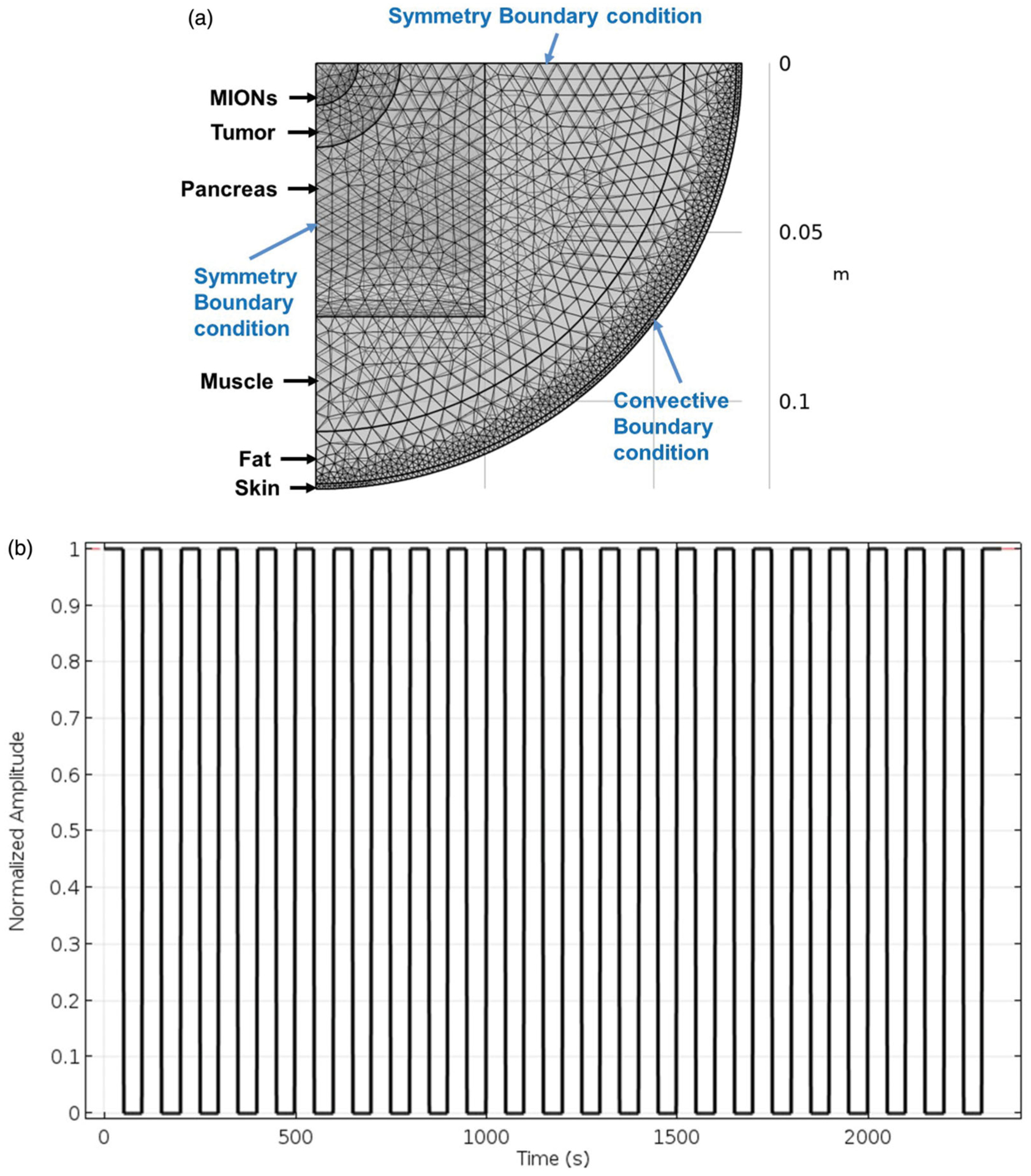
(a) Schematic of the meshed human scale computational model consisting of skin, fat, muscle, pancreas, tumor and magnetic iron-oxide nanoparticles (MIONs) embedded tumor with boundary conditions. (b) Sample pulsed alternating magnetic field (AMF) for a 50% duty-cycle. $\text{\% Duty }=\frac{\text{ Pulse ON time }}{\text{ Pulse ON time }+\text{ Pulse OFF time }}\times 100\text{\%}$.

To model heat transport in living tissues we used the Pennes bioheat equation formulated in the commercially available finite element software (COMSOL, Burlington, MA) (Equation (1)) [67]. We used a 3D multi-layer cylindrical model (Figure 2(a), Table 1) to study the effects of varied (a) spherical tumor size and, (b) duty-cycle of the pulsed AMF to reduce eddy current heating:

(1) $$\rho_{i}c_{i}\frac{\partial T_{i}}{\partial t}=k_{i}\nabla^{2}T_{i}+\rho_{b}c_{b}\omega_{b,i}\left(T_{b}-T_{i}\right)+Q_{m,i}+Q_{\text{eddy}}+Q_{p}$$

where subscripts *i* and *b* represent tissue layers (*i* = 1 … *n*) and blood; respectively. *ρ*, *c*, *k*, *T*, *Q*_*m*_ denote the density, specific heat, thermal conductivity, local temperature, metabolic heat generation rate, and *t* is the heating time. The heating rate of tissue per unit volume due to eddy currents, *Q*_*eddy*_, is given as follows:

(2) $$Q_{\text{eddy}}=\frac{\sigma_{t}}{2}{\left(\pi \mu_{0}rfH\right)}^{2}$$

where μ_0_ is the permeability of vacuum, *H* is AMF amplitude, *f* is AMF frequency, *r* radius of the eddy current path, σ_*t*_ is the electrical conductivity of the idealized tissue, and *Q*_*p*_ is the heating rate per unit volume of the tumor due to nanoparticles. *ρ*_*b*_*, c*_*b*_, *ω*_*b,n*,_ *T*_*b*_ denote density, specific heat, perfusion rate, and temperature, of blood, respectively. The thermal and electrical properties of the tissues were derived from the IT’IS foundation database [68]. Homogeneous AMF distribution was assumed based on the previously reported Maxwell-type induction coil [61]. Constant blood perfusion and homogeneous tissue properties were assumed to reduce the non-linearity in the simulation. Normal pancreas and pancreatic tumors were modeled with the same tissue properties except for blood perfusion and metabolic activity. Blood perfusion and metabolic heating rate of the pancreatic tumor relative to the surrounding pancreas tissue were modeled based on previously reported imaging data [13]. Pancreatic tumor with uniformly distributed MIONs was modeled as a sphere at the center of the multi-layer cylindrical torso to consider a worst-case scenario for nanoparticle heating, that is, minimizing contribution from eddy current heating in the tumor region. A uniform distribution of MIONs in half the tumor volume was considered as a constant volumetric heating rate to represent an image-guided MION injection. The nanoparticle heating rate was modeled based on the continuous polynomial approximation as a function of AMF amplitude (*H*) previously reported [69] for AMF frequency (*f*) of ~155 kHz. Tumor volume normalized MION dose of $5\frac{\text{mg Fe}}{cm^{3}\text{ of tumor }}$ and fixed AMF conditions $\left(H\times f<4.85\times 10^{8}\frac{A}{m\times s}\right)$ were used to study the role tumor size on the achievable temperatures in the tumor.

The role of pulsed AMF, that is, duty cycles, to reducing eddy current heating was based on previously reported small animal study [57]. The duty cycle was calculated using:

(3) $$\text{\% Duty }=\frac{\text{ Pulse ON time }}{\text{ Pulse ON time }+\text{ Pulse OFF time }}\times 100\text{\%}$$

The goal of this computational study was to understand how to minimize the nonspecific eddy current heating without affecting the thermal dose deposited in the tumor. Clinically challenging scenarios were simulated by using an AMF setting above the permissible limits, that is, $f>4.85\times 10^{8}\frac{A}{m\times s}$. Constant tumor size was used to investigate the role of varied duty cycles to minimize nonspecific heating. The maximum temperatures achieved in superficial tissue layers due to nonspecific heating and tumor due to MION heating were compared for 100, 50, 33.3 and 25% duty-cycles. A pulse train for 50% duty-cycle is shown in Figure 2(b). Mesh and time-step dependence studies ensured that calculated temperatures were sufficiently independent of a chosen model grid size and time step for the transient heating process.

## Results

### Small animal testing

**Figure 3.**
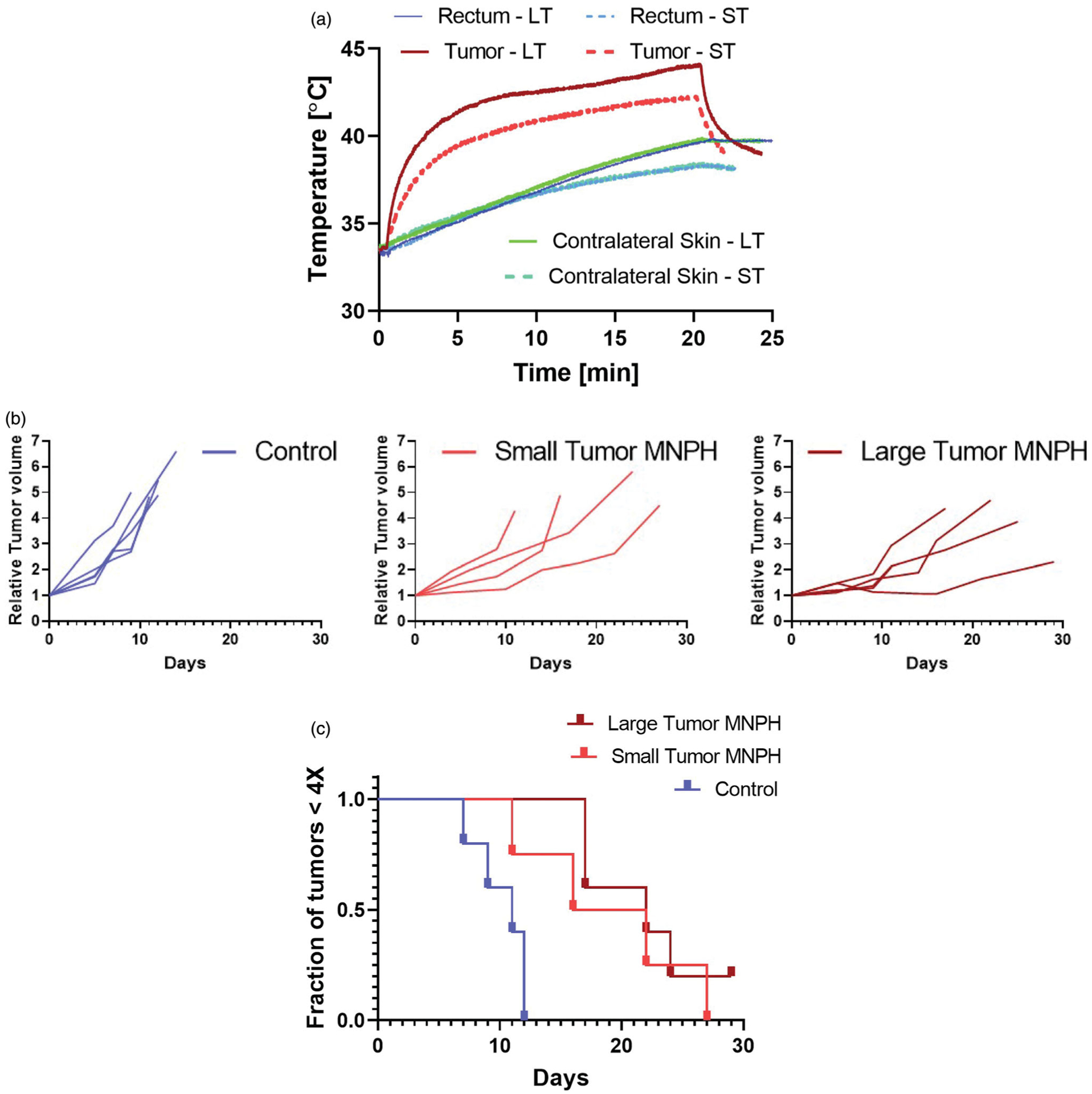
(a) An example of temporal temperature rise for small and large tumors during a MNPH treatment in MiaPaCa02 mice model. (b) Relative tumor growth curves for induvial mice. (c) Kaplan–Meier plot showing the outcome of MNPH treatment for untreated control, small and large tumors.

Measured maximum MiaPaCa02 intra-tumor temperatures for a 20 min AMF exposure are shown in Figure 3(a). The maximum temperature/CEM43 values for small and large injected tumors with volume normalized Fe dose was 42.3 °C with corresponding CEM43 of 2 min, and 44.1 °C with CEM43 of 15 min. Post MNPH treatment a slight discoloration of the skin of tumors was observed in many mice. The skin of some large tumors presented with drying scabs consistent with the higher temperatures measured in the tumor. Relative tumor growth delay for each animal are shown in Figure 3(b). MiaPaCa02 tumors responded favorably to tumor volume normalized iron dosed MNPH treatment for both the small and the large tumors (Figure 3(c)). The tumor growth delays were statistically significant (*p* < 0.005) for both treatment groups, relative to controls. Conversely, tumor growth between the small and large tumor MNPH treatment groups was not statistically significant (*p* = ~0.05).

### Computational simulations

**Figure 4.**
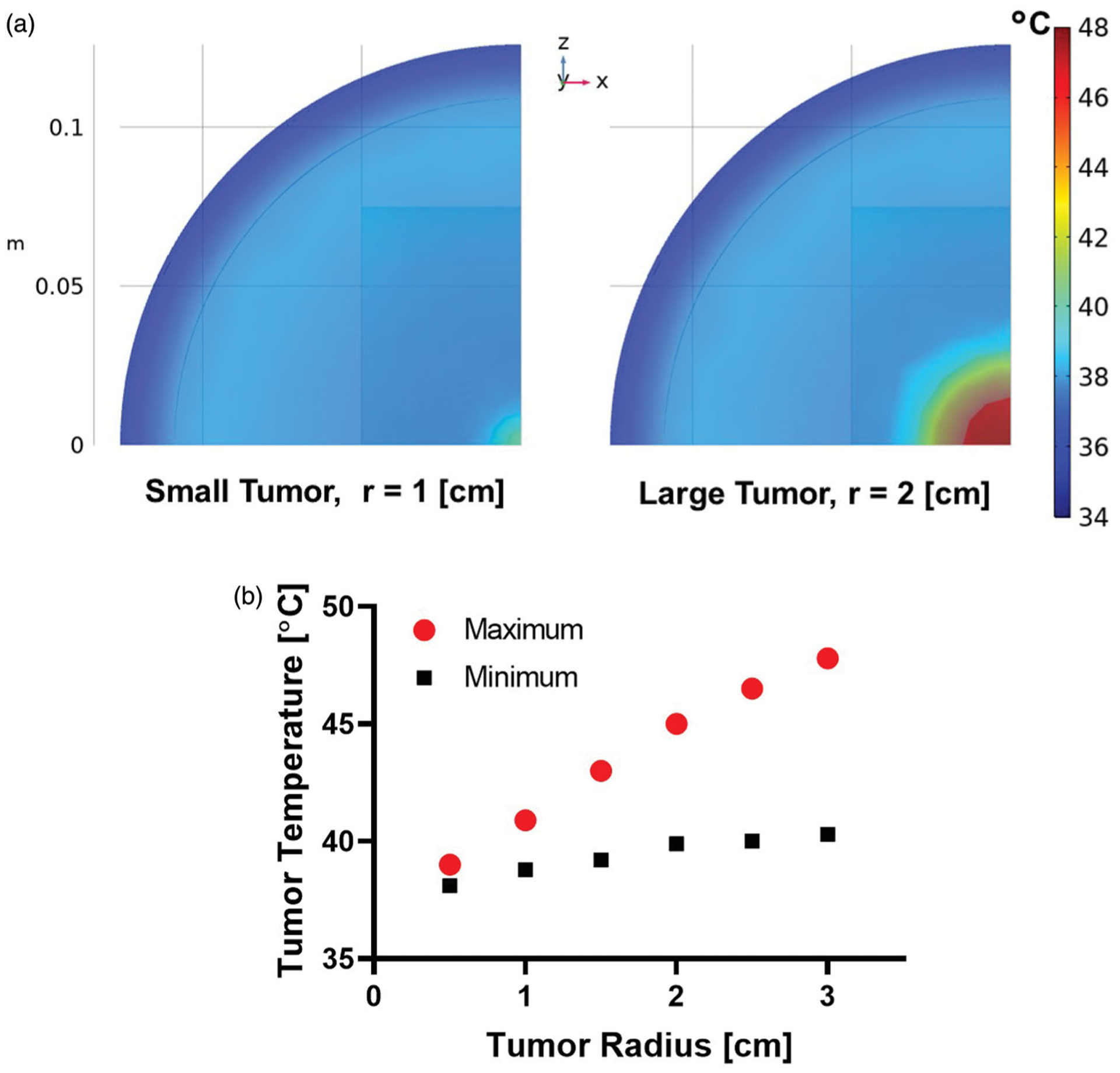
(a) Representative temperature distribution for tumor sizes with spherical radius of 1 cm and 2 cm, and (b) maximum and minimum tumor temperature as a function of tumor radius in human scale computational model after 20 min of MNPH treatment.

Temperature distribution at the end of 20 min heating for a tumor volume normalized MION dose of 5 mg Fe/cm^3^ of the tumor and fixed AMF conditions $\left(H\times f<4.85\times 10^{8}\frac{A}{m\times s}\right)$ for tumor radii of 1 and 2 cm are shown in Figure 4(a). The maximum tumor temperature increased as a function of spherical tumor radius (Figure 4(b)) because the total mass of MIONs delivered to the tumor also increases with tumor volume-normalized dose. Measured tumor temperatures in the MiaPaCa02 xenograft model also showed higher temperatures in large tumors compared to small tumors (Figure 3(a)). Thus, when the MION dose is normalized to tumor volume, MNPH removes the challenge of tumor size to the control of energy deposition by compensating with increased heating capability.

**Figure 5.**
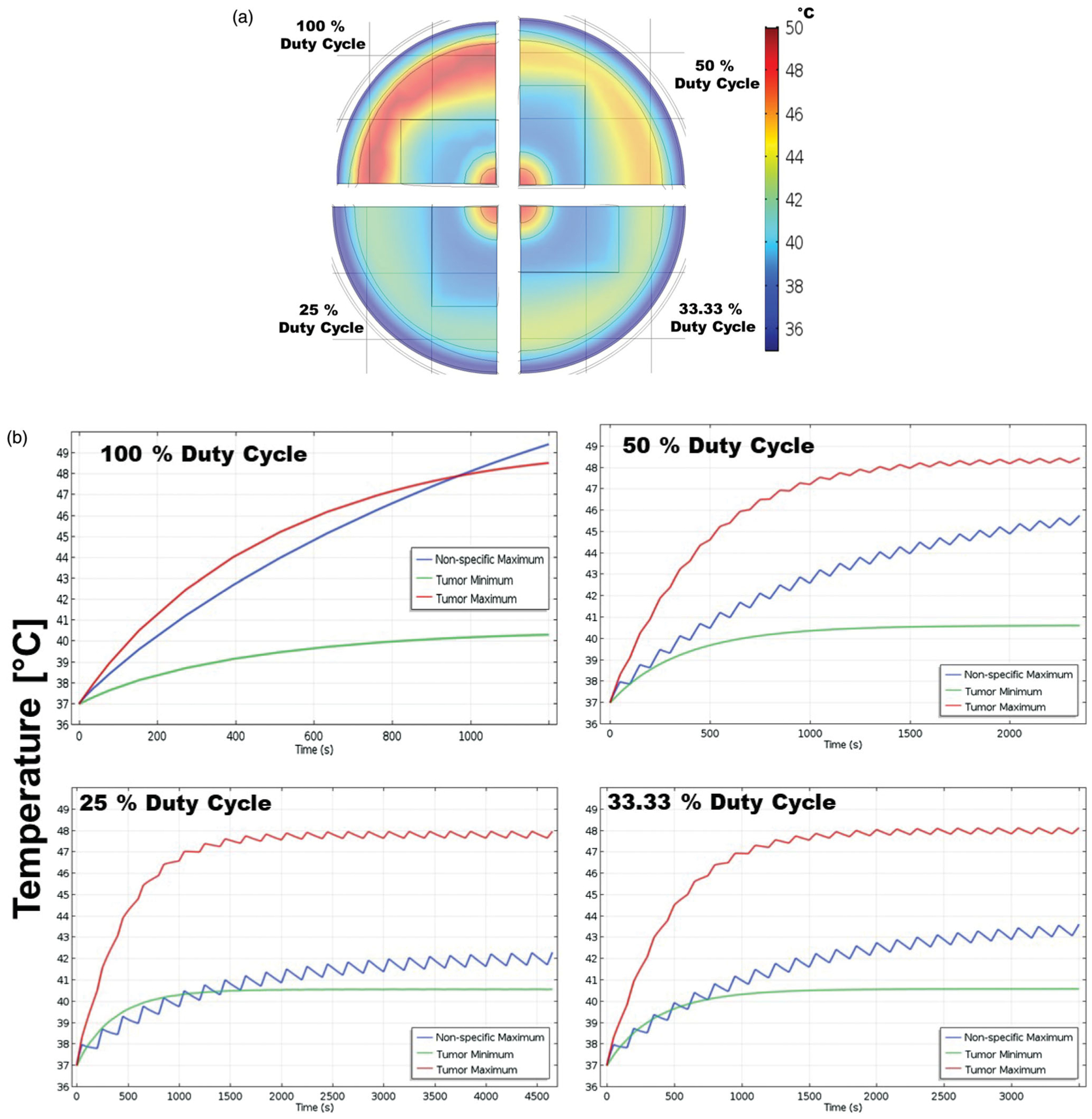
(a) Temperature distribution for 100%, 50%, 33.3% and 25% duty cycles in human scale computational model after 20 min of MNPH treatment. (b) Temporal temperature rise for 100%, 50%, 33.3% and 25% duty cycles in human scale computational model during the MNPH treatment. Note the change in the time scale on *x*-axis.

Temperature distributions for a total AMF-on time of 20 min at constant AMF conditions, $H\cdot f>4.85\cdot 10^{8}\frac{A}{m\cdot s}$, and constant tumor size with MION dose of 5 mg Fe/cm^3^ of the tumor for 100, 50, 33.3 and 25% duty-cycles are shown in Figure 5(a). Temperatures above 45 °C were predicted in the case of 100% duty-cycle due to eddy current heating. Muscle temperature rise due to eddy currents was minimized with reducing duty-cycle. Muscle temperature of ~41 °C was calculated for a 25% duty-cycle. Temporal profiles of the maximum tumor temperature, minimum tumor temperature and maximum temperature in muscle due to nonspecific eddy current heating for 100, 50, 33.3 and 25% duty-cycles are shown in Figure 5(b). The minimum tumor temperature and maximum tumor temperature changed little with the duty cycle. The maximum muscle temperature due to nonspecific eddy current heating, as expected showed a strong dependence on the duty-cycle. The maximum muscle temperature drastically changed from ~49.5 to ~42 °C when the duty-cycle was reduced from 100 to 25% (Figure 5(b)), even with treatment time increasing from 20 to 80 min to achieve the same average power. The minimization of eddy current heating with duty-cycle in human scale simulations follows the trend observed in mouse models [51].

## Discussion

Clinical approaches to treat BRPC and LAPC are poorly defined [3]. Large tumor volumes adversely affect progression-free survival and overall survival in patients with LAPC, leading to a poor prognosis [32,34–37]. Aggressive preoperative therapies such as SBRT and IMRT debulk the tumor to reduce the involvement of adjacent critical structures and improve the likelihood of potentially curative surgical resection, but associated risks complicate treatment decisions for effective disease control of large tumors [22–26,33]. Hypoxic conditions within the LAPC tumor microenvironment tend to make these tumors refractory to CRT and increase metastatic potential [45,66,70,71]. Hyperthermia can decrease intra-tumor interstitial fluid pressure which in turn can improve tumor blood flow and oxygenation [40,41,68,69,72].

Minimal thermal dose requirements are often difficult to achieve in deep tumors because of challenges to control energy deposition. MNPH offers the potential to address this challenge because the MIONs, embedded within the target, heat from within and the total amount of energy deposited depends on the amount of MIONs embedded in the tumor. AMF power modulation controls the heat produced by the MIONs, thereby providing energy control with appropriate temperature data. Furthermore, multiple MNPH heat treatments can be performed after a single image-guided injection of MIONs, because they tend to remain within the tumor [49–51,54].

We sought to ascertain in a pilot mouse study and human-scale computer simulations if MNPH offers the potential to reduce the effect of tumor volume for treatment. The motivating rationale is that for MNPH, the thermal dose deposited in the tumor depends on several interdependent variables – the total MION dose, their heating rate, and AMF parameters (*H* × *f*). Certainly, there are additional biological and environmental variables, for example, tumor type, size-dependent physical properties of the tumor, injection rate and volume, etc.; however, total heat energy deposited into a tumor ultimately depends on the power and duration of heating. With increased nanoparticle content, there is increased power given all other conditions are fixed. We hypothesized that MNPH reduces the influence of tumor size on treatment outcome by enabling a tumor volume-normalized increase of heating power. While the thermal rationale for such an approach is straightforward, the biological effects may be complex. Further, when scaling to human sizes nonspecific heating from AMF-induced eddy currents becomes a challenge. Therefore, we tested this hypothesis in two separate but related experiments: (a) a pilot mouse study using two different tumor volumes to test effects of volume-normalized MNPH on tumor growth; and, (b) computational studies accounting for human-scale tissues and tumors, with eddy current heating.

Tumor growth of subcutaneous human MiaPaCa02 xenograft tumors demonstrated no statistical difference between small and large tumors when treated with MNPH using a tumor volume normalized MION dose. The iron concentrations in small (0.15 cm^3^) and large (0.3 cm^3^) tumors were 0.83 mg Fe and 1.7 mg Fe, respectively (Table 2). Correspondingly, a higher tumor temperature was measured in large tumors compared to small tumors (44.1 vs. 42.3 °C) (Figure 3(a)), leading to an expected therapeutic effect.

While these pilot results are encouraging, we note significant limitations with both the mouse model and numbers in the study. Small animal models provide a limited range for studies of volume-dependent effects. To acquire sufficient tumor growth data for comparing between the groups, selection of initial tumor volume(s) was limited to <0.5 cm^3^, which imposed serious study design limitations. Intratumor injections of MIONs become unreliable for tumor volumes <0.1 cm^3^ further narrowing the size range of tumors for study. Within these limits, we selected tumor volumes separated by a two-fold variation to assess the potential for therapeutic effects of volume-normalized MNPH. More rigorous testing of this hypothesis and further optimization of this approach will require relevant large, scale-models. Besides companion animals or humans, no relevant large-tumor models are readily available necessitating computational modeling to test the physical assertions of the hypothesis.

Human-scale simulations predicted similarly higher temperatures in large tumors with volume-normalized MNPH (Figure 4(a,b)). This is expected given results reported from computational and experimental studies of size-dependent effects using other technologies [73–75]. From the perspective of physics, sustaining a thermal dose in smaller tumors with MNPH can be challenging [73]. Heat loss to the surrounding tissue mediated by thermal conduction is higher for smaller tumors due to the high surface area per unit volume compared to a large tumor. Provided the MION dose is adjusted for tumor volume, MNPH favors treating large tumors compared to other hyperthermia technologies that deposit/focus energy using external applicators because the MNPH heat source(s) are embedded within the tumor.

While computational models provide valuable insight, several model limitations reduce the clinical relevance of the results. Uniform MION distribution in the tumor was assumed instead of a more realistic or non-uniform distribution to reduce the non-linearity of the computational model. The role of various MION distributions based on the histology [57] to deliver the required thermal dose was extensively studied in the context of AMF power modulation [56]. Future efforts will benefit from pretreatment imaging of the MION distribution in tissues, ideally with a compatible imaging modality such as magnetic particle imaging (MPI) [76].

In the computational studies, we considered (constant) blood perfusion due to the microvasculature and small vessel structures, and ignored the heat sink effect from large blood vessels to reduce computational complexity. LAPC tumors generally exhibit low blood flow, thus making MNPH better suited for favorable treatment(s) [13]. Nevertheless, the degree to which relative tumor and blood flow parameters affect thermal dose and treatment outcome requires further evaluation. Future studies should also consider convective cooling due to large blood vessels with diameter >3 mm, to simulate realistic clinical scenarios, such as involvement with major mesenteric vessels in LAPC.

Nonspecific eddy current heating is a limiting factor for the translation of MNPH to humans to treat deep tissue tumors [56,59]. Pulsed AMF was successful to manage and limit excessive nonspecific heating in mouse models [62]. This approach however is not integrated into clinical MNPH. The degree to which pulsing AMF power can improve the ratio of nanoparticle heating to eddy current heating in clinically unfavorable conditions such as AMF exposure above clinically permissible limit $H\times f>4.85\times 10^{8}\frac{A}{m\times s}$ and relatively low MION concentration of 5 mg Fe/cm^3^ of the tumor was tested in human-scale computational models [51–55]. Pulsed AMF is easier to implement in clinical situations compared to the relative tissue displacement method previously proposed [59]. Results of simulations demonstrate significant potential to enhance MNPH with AMF pulsing because blood perfusion in normal tissues more effectively dissipates the heat during AMF off cycles. To achieve the equivalent thermal dose, treatment duration must be adjusted to compensate for the reduced duty with MNPH. These results are encouraging, but limited, by the assumptions and simulation parameters that included constant blood perfusion, homogeneous tissue properties, simple geometries, and a simplistic duty cycle modification. Advanced multi-point temperature feedback-control algorithms with more complex AMF power modulation could be used to reduce the treatment times to clinically acceptable levels (≤60 min) while minimizing the nonspecific eddy current heating [56]. Such temperature-controlled AMF power modulation approaches have also shown the potential to compensate heterogeneous nanoparticle distributions in the tumor.

In summary, we propose modulated power heating of MIONs using temperature feedback to minimize eddy current heating and to address principal challenges of MNPH. Additional studies are required to understand the biological consequences of tumor size in the context of combined MNPH and CRT, taking into account MION spatial heterogeneity in the tumor. Future studies should be directed toward the use of patient image-based geometries with temperature-dependent tissue properties to develop treatment-planning systems for MNPH with multi-point thermometry. In the long-term, wider acceptance of MNPH in a clinical application depends on a clear demonstration that its inclusion significantly enhances treatment response and patient survival.

## Conclusions

Tumor volume normalized MNPH offers the potential to reduce the adverse effect of tumor size on treatment outcome, as predicted by heat transfer simulations. Results obtained from pilot mouse studies demonstrated modestly higher temperatures were achieved and sustained in larger tumors, however, this did not lead to significant tumor growth inhibition. Inherent limitations of small animal models for such studies and spatial heterogeneity of MIONs in tumors continue to present challenges. From the physics of heat transfer and computational modeling, it is clear that large tumors are candidates for this approach with MNPH due to the high MION dose and low heat conduction mediated loss to the surrounding tissue compared to small tumors. Pulsed AMF presents a promising approach to enhance MNPH in challenging clinical situations by minimizing nonspecific eddy current heating without sacrificing thermal dose to the target, provided accurate spatial thermometry, and treatment duration is adjusted. Further computational studies with human voxel models and experimental studies using tissue-equivalent gel phantoms and large animal models are required to rigorously test the potential for pulsed AMF applications and refine them for the clinic. Successful clinical translation of this approach will facilitate the treatment of challenging locally advanced pancreas cancer.

## Figures and Tables

**Table 1.** Summary of the computational domain geometry.

	Shape	Axis	Outer radius (m)	Height (m)
Pancreas	Cylinder	*X*	0.075	0.1
Muscle	Cylinder	*Y*	0.109	0.55
Fat	Cylinder	*Y*	0.1244	0.55
Skin	Cylinder	*Y*	0.126	0.55
Tumor	Sphere	*Z*	0.005–0.03	NA
MION	Sphere	*Z*	Radius: 0.5 × tumor radius	NA

**Table 2.** Summary of treatment parameters.

S. No.	Initial tumor volume (cm^3^)	Injected material	Injected volume (μl)	Injected Fe [mg]	AMF exposure	Purpose
1	0.16	MION	37	0.87	NO	Growth delay
2	0.16	MION	38	0.89	NO	Growth delay
3	0.16	MION	37	0.87	NO	Growth delay
4	0.16	MION	36	0.85	NO	Growth delay
5	0.19	MION	44	1.04	NO	Excluded - tumor volume outside the range
6	0.15	PBS	34	0	YES	Growth delay
7	0.15	MION	35	0.82	YES	Temperature measurement
8	0.13	MION	31	0.73	YES	Growth delay
9	0.15	MION	34	0.8	YES	Growth delay
10	0.13	MION	30	0.71	YES	Growth delay
11	0.14	MION	32	0.75	YES	Died - post treatment
12	0.16	MION	38	0.89	YES	Growth delay
13	0.16	MION	37	0.87	YES	Temperature measurement
14	0.32	MION	75	1.77	YES	Growth delay
15	0.30	MION	69	1.62	YES	Growth delay
16	0.27	MION	64	1.51	YES	Growth delay
17	0.31	MION	72	1.7	YES	Growth delay
18	0.33	MION	77	1.81	YES	Growth delay
